# Reflecting on the *Communications Biology* Editor Training Program

**DOI:** 10.1038/s42003-024-06005-y

**Published:** 2024-03-19

## Abstract

In 2023, we conducted a virtual program to introduce early-career researchers to the fundamentals of academic journal editing. Here, we reflect on the mission of this program and its outcomes.

When interacting with researchers at conferences or institutional site visits, our editors are often asked about how they prepared for a career in publishing, and whether there are any programs that might serve as an entry-point for an editorial career. These questions are increasingly relevant considering the limited number of available tenure-track research positions compared to the number of PhD graduates each year. As outlined in our editorial values statement, part of our mission as a journal is to “engage with researchers at all stages of their career to understand their needs and advocate for positive change”. In that spirit, in 2023 we developed an Editor Training Program to provide hands-on experience and a personalized training environment for ECRs interested in editorial careers.

Briefly, the Editor Training Program consisted of hour-long virtual sessions that took place over six consecutive weeks, with each week focusing on a different topic or task within the publication process (Table 1). After each session, participants were given take-home assignments meant to simulate specific editorial tasks like assessing a new manuscript or evaluating reviewer comments. Participants were also paired with one of our in-house editors, who provided direct feedback on each assignment. The last session of the program was a career panel featuring our external Editorial Board Members and editors at other Nature Portfolio journals, with the aim of exposing trainees to other editorial career paths. Finally, we invited each trainee to write a Research Highlight that would be published in *Communications Biology*. These Research Highlights^1–8^ can be found on our journal website.

**Table 1:** *Communications Biology* Editor Training Program Schedule

Program Week	Topic	Take-Home Assignment
1	Introduction to editing and academic publishing	Review an Article
2	Discussing the roles of editors vs. reviewers	Write an editorial assessment for an Article
3	Identifying potential reviewers and evaluating their feedback	Write an editorial assessment for an Article, and draft a post-review decision based on reviewer feedback.
4	Assessing journal front-half content and writing Research Highlights	Write editorial assessment for a Review, Mini-Review, Comment, or Perspective. Identify 3 articles for Research Highlight.
5	The editorial process after accepting a manuscript for publication	Work on Research Highlight.
6	Editorial career panel	Finalize Research Highlight

We first ran this program as a pilot between July-August 2022, with a cohort of 5 trainees split across the USA (3), Spain (1), and Italy (1). For the purpose of this pilot, we invited participants we had previously met through editorial outreach events, and trainees were similarly given the chance to complete a Research Highlight^9–13^. In 2023, however, we hosted an open call for participants, which asked applicants to complete 3 short essay questions:

1. Tell us why participating in this training program is important to you.
2. Provide a brief, lay summary of your research (using terms that non-specialists could understand).
3. Describe a recent paper (published since January 2022) in your area of expertise and why it is important to your field (using terms which non-specialists can understand). If you were to receive this paper as an editor, what expertise would you look for in editors?

We ultimately considered 158 applicants from 40 countries, with most applications coming from India (17.6%), the USA (14.4%) or Germany (7.8%), though the full distribution can be seen in Fig. 1. Applications were judged by a panel of in-house editors and our Editorial Board Members, to recruit a final cohort of 8 participants spread across India (2), the USA (1), Spain (1), Japan (1), Canada (1), Belgium (1), and Australia (1). At the end of the program, we received feedback from 7/8 of these participants, all of whom agreed that the experience provided “new information about the publication process”, that it helped them “feel confident about what an editorial career entails”, and that they would “recommend the program to a colleague” (based on a score of 4 or more, on a scale of 1–5). In the words of one trainee:

“Confidence is the most succinct way to describe what I feel I’ve gained. I loved getting insight into the publication process from the editorial perspective.”

**Fig. 1:**
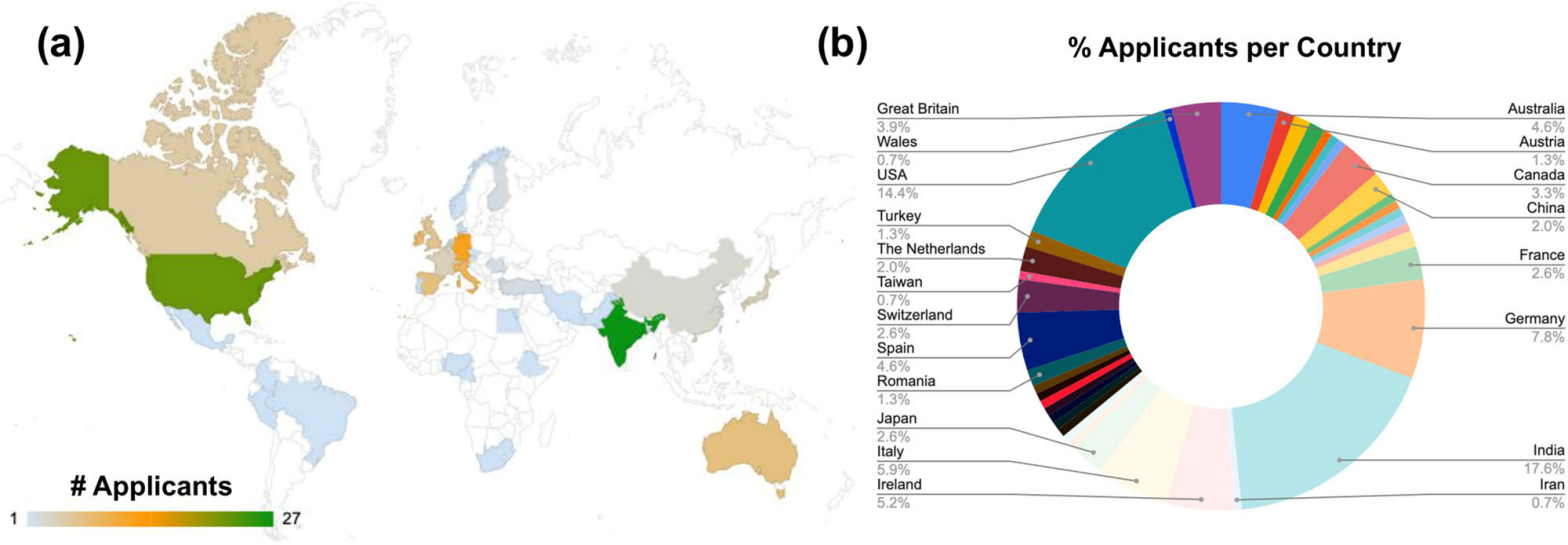
Geographic distribution of 158 applicants for the *Communications Biology* Editor Training Program in 2023. **a** World map depicting the number of applicants per country. **b** Pie chart depicting the percentage of total applicants per country.

Conducting this program has been a rewarding experience for our editorial team, and we hope to provide similar career development experiences for trainees in the future. After all, in the words of one of our trainees: “Workshops like this are such an integral part of academia, as is the actual editing process and more researchers should have the opportunity to learn about it.”
